# Developing a novel risk prediction model for severe malarial anemia

**DOI:** 10.1017/gheg.2017.8

**Published:** 2017-09-11

**Authors:** E. B. Brickley, E. Kabyemela, J. D. Kurtis, M. Fried, A. M. Wood, P. E. Duffy

**Affiliations:** 1 Laboratory of Malaria Immunology and Vaccinology, Division of Intramural Research, National Institute of Allergy and Infectious Diseases, National Institutes of Health, Rockville, Maryland, USA; 2 Department of Public Health and Primary Care, University of Cambridge, Cambridge, UK; 3 Department of Epidemiology, Geisel School of Medicine at Dartmouth College, Lebanon, New Hampshire, USA; 4 Muheza Designated District Hospital, Muheza, Tanzania; 5 Department of Pathology and Laboratory Medicine, Rhode Island Hospital, Brown University Medical School, Providence, Rhode Island, USA

**Keywords:** Anemia, biomarkers, cytokines, malaria, personalized medicine, risk prediction

## Abstract

As a pilot study to investigate whether personalized medicine approaches could have value for the reduction of malaria-related mortality in young children, we evaluated questionnaire and biomarker data collected from the Mother Offspring Malaria Study Project birth cohort (Muheza, Tanzania, 2002–2006) at the time of delivery as potential prognostic markers for pediatric severe malarial anemia. Severe malarial anemia, defined here as a *Plasmodium falciparum* infection accompanied by hemoglobin levels below 50 g/L, is a key manifestation of life-threatening malaria in high transmission regions. For this study sample, a prediction model incorporating cord blood levels of interleukin-1β provided the strongest discrimination of severe malarial anemia risk with a C-index of 0.77 (95% CI 0.70–0.84), whereas a pragmatic model based on sex, gravidity, transmission season at delivery, and bed net possession yielded a more modest C-index of 0.63 (95% CI 0.54–0.71). Although additional studies, ideally incorporating larger sample sizes and higher event per predictor ratios, are needed to externally validate these prediction models, the findings provide proof of concept that risk score-based screening programs could be developed to avert severe malaria cases in early childhood.

## Introduction

Although improved uptake of vector control measures and first-line antimalarial medication has reduced the number of deaths due to malaria in children younger than 5 years of age by nearly 60% since the year 2000, malaria still remains the fourth leading cause of child death in sub-Saharan Africa [1, 2]. In the *Global Technical Strategy for Malaria 2016–2030* endorsed in May 2015, the World Health Assembly set the goal of reducing the 2015 malaria mortality rates by a further 90% by 2030 [3]. Mathematical models of *Plasmodium falciparum* transmission suggest that scaling-up of existing population-wide interventions, including through expanded access to long-lasting insecticidal nets (LLINs), seasonal malaria chemoprevention in children aged 6 months to 5 years, and artesunate therapies, could reduce malaria mortality rates by up to an estimated 74% (95% CI 67–84%) [4]. Further, the incorporation of ‘near-term innovations,’ such as LLINs with four-year half-lives, broader use of seasonal malaria chemoprevention in children aged 6–10 years, and the use of alternative chemopreventive medications in East African regions with high sulfadoxine-pyrimethamine parasitic resistance, could lead to mortality reductions of up to 81% (95% CI 76–87%) [4]. By using population-level strategies that seek to control the causes of malarial death, the proposed scenarios could potentially save millions of lives. Nevertheless, implementation of these agendas will be highly costly and, barring development of the near-term innovations, will fall short of achieving the 90% mortality reduction benchmark. Therefore, we asked: Would it be feasible to complement the ‘population approach’ with a ‘high-risk approach’ that aims to provide additional targeted intervention to the children who are most susceptible to developing life-threatening malaria? [5]

Using data collected at the time of birth as part of the Mother Offspring Malaria Study (MOMS) Project birth cohort in Muheza, United Republic of Tanzania, between 2002 and 2006, we sought to develop a prognostic model for severe malarial anemia as a pilot study for investigating whether personalized medicine approaches could have value for the reduction of malaria-related mortality. In regions with high malaria transmission, severe malarial anemia, here defined as a *P. falciparum* infection with hemoglobin concentrations below 50 g/L, is the most common manifestation of life-threatening malaria in young children [6]. Moreover, previous studies on this cohort provided evidence that cord levels of the pro-inflammatory cytokines tumor necrosis factor (TNF) and interleukin-1β (IL-1β) may correlate with children's cytokine levels throughout infancy and may be inversely related to future parasite burdens and severe malarial anemia risk [7, 8]. Building on this research, we aimed to: (i) assess the discriminative ability of subject characteristics and biological markers measured at delivery for the prediction of a child's probability of developing severe malarial anemia during the first 3 years of life and (ii) estimate the potential clinical benefit of using a risk prediction model to guide further intervention.

## Methods

### Study cohort

The study setting, location, dates, and methods for data collection and follow-up in the MOMS Project have been described in detail previously [9]. To be eligible for the study, children had to be: (i) born to human immunodeficiency virus negative mothers, (ii) sickle cell disease free, (iii) singleton births, and (iv) followed for a minimum of 28 days. The primary outcome was time to first severe malarial anemia. Clinical teams monitored children for severe malarial anemia at all sick visits and during routine visits occurring every 2 weeks in infancy and every 4 weeks thereafter until age 4 or study termination. Parasitemia was assessed by Giemsa-stained thick blood smear, and hemoglobin levels were measured with an impedance-based analyzer. The candidate predictors included: sex, genotypes for beta-globin (i.e., AA and AS) and alpha-thalassemia (i.e., *α*/*α*, *α*/*α* − 3.7, and *α* − 3.7/*α* − 3.7), birth weight, maternal age, gravidity (i.e., here indicated by number of previous pregnancies), transmission season at delivery (i.e., early high season (May–July), late high season (August–October), early low season (November–January), and late low season (February–April), placental malaria status at delivery (i.e., the detection of *P. falciparum* parasitemia in placental blood collected through mechanical pressing of full-thickness tissue), household bed net possession at delivery (i.e., treated, untreated, and no/unknown), intermittent preventive treatment in pregnancy (IPTp) (i.e., 0 versus 1+ doses), and a panel of cord blood cytokines and receptors listed in Table 1 and visualized in Supplementary Fig. S1. Sickle cell trait was determined by cellulose acetate paper electrophoresis (Helena Laboratories, Beaumont, TX, USA). Alpha-thalassemia was determined by PCR [10]. Birth weight was evaluated within 24 h after delivery. Data on maternal age, gravidity, household bed net possession, and IPTp dosage were self-reported. Soluble cytokines and receptors in cord blood plasma were measured using commercially available multiplex, bead-based platforms (BioPlex^®^, BioRad, Irvine, CA, USA) and custom-made assay kits as previously described [11]. Samples that did not produce detectable concentrations of a given marker were assigned a value of half the limit of detection of that marker. As the numbers of measurements detectable by immunoassays were low for interleukin-4 (9.7%, *n* = 76) and interferon-γ (20.6%, *n* = 161), these cytokines were evaluated as binary variables (i.e., detectable levels versus levels below the limits of detection); other cord cytokines and receptors were log_e_-transformed to obtain approximately normal distributions (Supplementary Fig. S1).

**Table 1.** Distribution of baseline characteristics in the MOMS Project (2002–2006) birth cohort in Muheza, Tanzania

Characteristic	Total	*n*, % or median (IQR)
Sex	880	
Female		423, 48.1%
Male		457, 51.9%
Beta-globin genotype	859	
AA		718, 83.6%
AS		141, 16.4%
Alpha-thalassemia genotype	822	
*α*/*α*		389, 47.3%
*α*/*α* − 3.7		336, 40.9%
*α* − 3.7/*α* − 3.7		97, 11.8%
Birth weight, kg	880	3.2 (2.9, 3.5)
Maternal age, years	880	25 (20, 30)
Number of previous pregnancies	880	1 (0, 3)
Transmission season at birth	880	
Early high transmission season		191, 21.7%
Late high transmission season		240, 27.3%
Early low transmission season		230, 26.1%
Late low transmission season		219, 24.9%
Placental malaria	880	
Placental malaria-negative		765, 86.9%
Placental malaria-positive		115, 13.1%
Bed net possession at enrollment		
Treated bed net-positive		111, 12.6%
Untreated bed net-positive		344, 39.1%
Bed net-negative/ unknown		425, 48.3%
Intermittent preventive treatment in pregnancy	820	
0 doses		107, 13.1%
1+ doses		713, 87.0%
Tumor necrosis factor, pg/mL	781	121 (69.4, 182)
Tumor necrosis factor – receptor I, pg/mL	781	2160 (1480, 2880)
Tumor necrosis factor – receptor II, pg/mL	781	472 (323, 696)
Interleukin – 1β, pg/mL	781	6.00 (3.00, 11.5)
Interleukin – 4	781	
Below the limit of detection		705, 90.3%
Detectable		76, 9.7%
Interleukin – 5, pg/mL	781	2.60 (1.00, 5.21)
Interleukin – 6, pg/mL	781	7.00 (2.31, 18.5)
Interleukin – 10, pg/mL	781	3.52 (1.51, 6.04)
Interferon – γ	781	
Below the limit of detection		620, 79.4%
Detectable		161, 20.6%

### Study oversight

This study was approved by the US National Institutes of Health International Clinical Studies Review Committee of the Division of Microbiology and Infectious Diseases. Ethical clearance was provided by the Institutional Review Boards of the Seattle Biomedical Research Institute and the National Institute for Medical Research in Tanzania. Participating mothers provided written informed consent for themselves and their newborn child. Prompt care was provided to sick children in accordance with Tanzanian Ministry of Health protocols. All subsequent laboratory measurements were performed on de-identified samples.

### Statistical analysis

Associations between the baseline risk markers and severe malarial anemia were quantified using Cox proportional hazard models. Candidate markers were investigated first individually using all of the available data for a given marker and then in combined analyses restricted to those individuals with complete information. A basic model using the pragmatic markers of sex, gravidity, transmission season at delivery, and bed net possession was also explored. Risk discrimination was assessed using Harrell's C-index, a generalization of the area under the receiver operating curve suitable for time-to-event analyses [12]. The C-index quantifies the ability of a model to correctly predict the order of disease events and ranges in value from 0.5 (i.e., chance) to 1 (i.e., perfect prediction of order failure). To investigate the magnitude of optimism (i.e., overfitting of the model due to the small number of cases and high number of predictors) [13], bootstrapping was performed using the technique originally described by Harrell *et al*. [14]. For this method, the data set was resampled to produce 200 replicated data sets. Then, the prognostic models were fitted to each of the 200 bootstrapped data sets and the original data set. The C-indices were calculated for all data sets, and the difference between each resampled data set and the original data set was recorded. Estimates of optimism were calculated as the average of the 200 differences. To correct the models, the optimism estimates were then subtracted from each of the original C-indices. To elucidate the extent to which risk prediction might have benefit for public health campaigns against malaria, a modeling exercise was performed to explore a hypothetical screening program with targeted versus randomized allocation of antimalarial intervention. Specifically, it was assumed that the population had 1000 children, an incidence of severe malarial anemia equivalent to that observed in the subset of the MOMS Project cohort with available cord blood cytokine measurements (*n* = 781) who were followed between 2002 and 2006, and resources available to administer an intervention with 75% protective efficacy [15, 16] to 200 of the children. All statistical analyses were performed using Stata – version 12 (StataCorp LP, College Station, TX, USA).

## Results

**Fig. 1.**
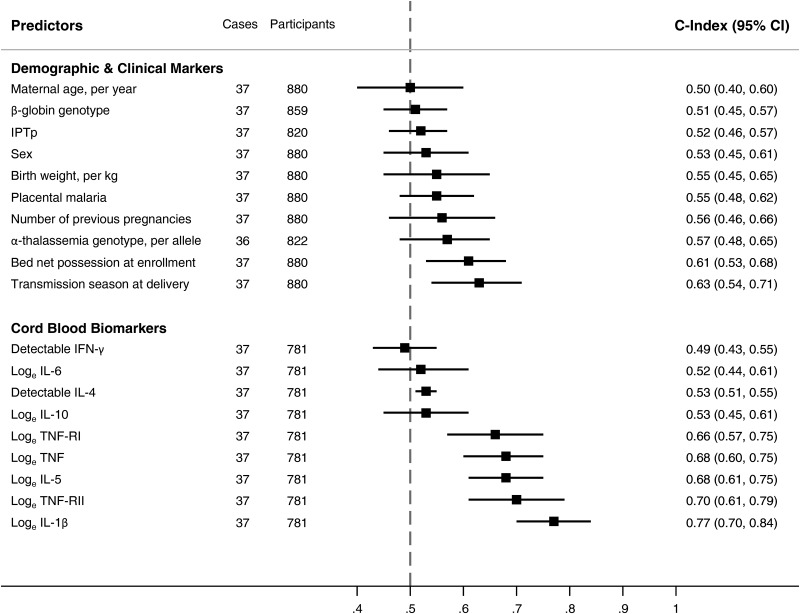
Discrimination of severe malarial anemia risk in univariate prognostic models in the MOMS Project (2002– 2006) birth cohort in Muheza, Tanzania, ranked in order of increasing C-index estimates. IPTP, intermittent preventive treatment in pregnancy; IFN, interferon; IL, interleukin; TNF-RI, tumor necrosis factor-receptor I; TNF, tumor necrosis factor; TNF-RII, tumor necrosis factor-receptor II.

**Fig. 2.**
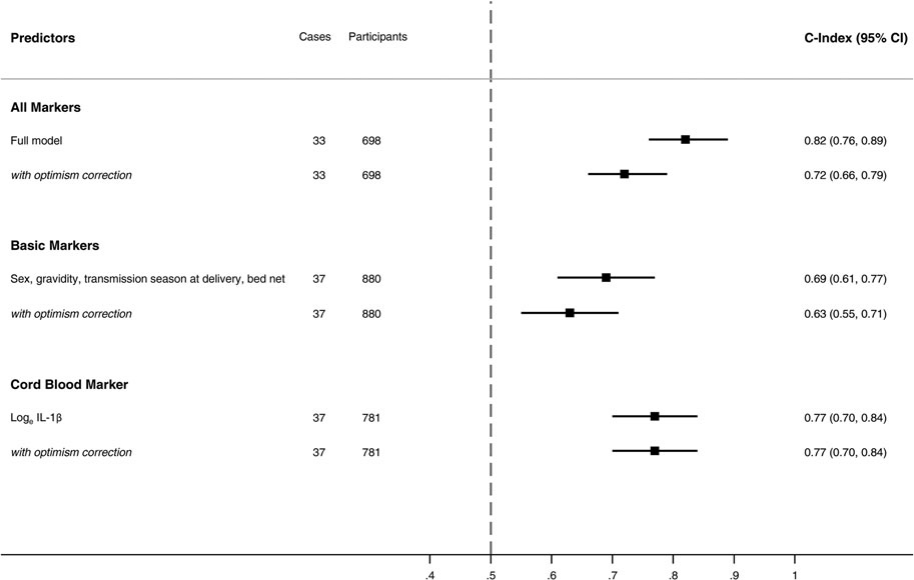
Optimism-corrected C-indices for severe malarial anemia risk in the MOMS Project (2002–2006) birth cohort in Muheza, Tanzania. Abbreviations: IL, interleukin.

During a median follow-up of 2.0 (interquartile range, 1.1–2.9) years, 37 incident severe malarial anemia events were recorded in the 880 children participating in the MOMS Project cohort. Out of the baseline demographic and clinical markers, the transmission season at delivery (evaluated quarterly) provided the best discrimination of severe malarial anemia risk, and its associated C-index was 0.63 (95% CI 0.54–0.71) (Fig. 1). Household bed net possession at enrollment was also significantly predictive in univariate models with a C-index of 0.61 (95% CI 0.53–0.68) (Fig. 1). Combining these two predictors with information on the child's sex and the mother's gravidity that are readily obtainable at the time of birth yielded an additive C-index of 0.69 (95% CI 0.61–0.77). However, the estimated optimism for this prognostic model was substantial, and after performing a bootstrap correction, the C-index remained significant but attenuated to 0.63 (95% CI 0.55–0.71) (Fig. 2).

**Fig. 3.**
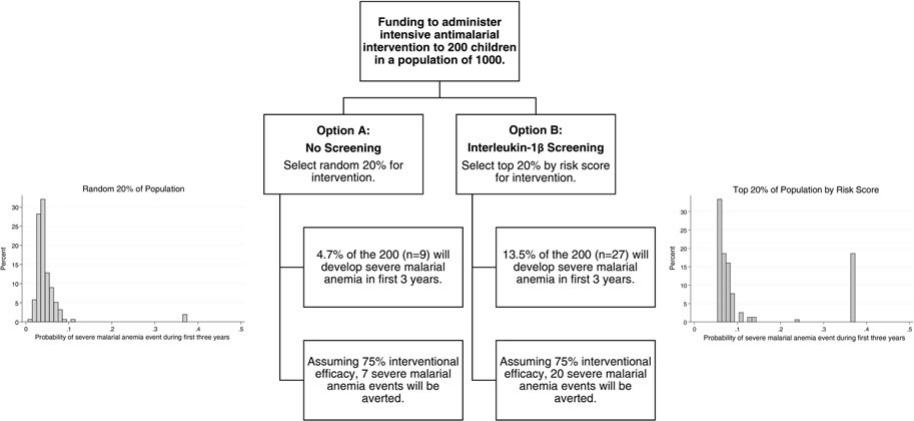
Schematic diagram for hypothetical interleukin-1β screening program with targeted allocation of antimalarial intervention.

Out of the cord blood markers, interleukin-1β yielded the strongest discrimination of risk with a C-index of 0.77 (95% CI 0.70–0.84) and had an average optimism of only 0.0044 (Figs 1 and 2). In a hypothetical cord blood screening program that measures interleukin-1β in 1000 children, the targeted allocation of an intensive antimalarial intervention with 75% efficacy to the top 20% of the population at risk of severe malarial anemia (i.e., the high risk group) would avert 13 additional severe malarial anemia events compared with the distribution of the intervention to a randomly selected sample of 20% of the population (Fig. 3). Stratifying the population's risk with this 20% cut-off yielded a ROC area of 0.69 (95% CI 0.61–0.78) (Table 2). Given the total population prevalence of severe malarial anemia of 4.7% (95% CI 3.4–6.5%), the positive predictive value (i.e., the probability that children who received the intensive intervention developed severe malarial anemia) was enriched to 13.5% (95% CI 8.5–19.8%) in the high risk group, and the negative predictive value (i.e., the probability that children who did not receive the intensive intervention did not develop severe malarial anemia) remained high at 97.4% (95% CI 95.9–98.5%) (Table 2). In this modeled scenario, the cord blood of 77 (i.e., 1000÷13) children would have to be screened for interleukin-1β to avert one additional severe malarial anemia case.

**Table 2.** Cord blood interleukin-1β risk scores for severe malarial anemia in the MOMS Project (2002–2006) birth cohort in Muheza, Tanzania (*n* = 781)

	Predicted probability of severe malarial anemia by risk score quintile (%)	No. of cases/no. in quintile	Sensitivity (%) (95% CI)	Specificity (%) (95% CI)	ROC area (95% CI)	Positive predictive value (%) (95% CI)	Negative predictive value (%) (95% CI)
Q1	0.5–3.1	0/160	–	–	–	–	–
Q2	3.1–3.7	3/153	100 (91–100%)	22 (19–25%)	0.61 (0.59–0.62)	6.0 (4.2–8.1%)	100 (98–100%)
Q3	3.7–4.3	4/156	92 (78–98%)	42 (38–46%)	0.67 (0.62–0.71)	7.3 (5.1–10%)	99 (97–100%)
Q4	4.4–5.3	9/156	81 (65–92%)	62 (59–66%)	0.72 (0.65–0.78)	9.6 (6.6–13%)	97 (96–99%)
**Q5**	**>5.3**	**21/156**	**57 (40–73%)**	**82 (79–85%)**	**0.69 (0.61–0.78)**	**13.5 (8.6–20%)**	**97 (96–99%)**

The total population prevalence of severe malarial anemia during the first 3 years of life was 4.7% (95% CI 3.4–6.5%). For estimation of sensitivity, specificity, ROC area, and positive and negative predictive value, the lower limit of each risk score quintile was used to define a ‘positive’ result of the screening test (e.g., Q4, 4.4% predicted probability or higher = positive). Bolded text indicates the performance of the high risk group cut-off (i.e., top 20%) used in the public health modeling exercise illustrated in Fig. 3.

## Discussion

These data provide proof of concept that a prognostic model based on measurements captured at the time of birth can be developed to facilitate the selection of high-risk children for additional targeted intervention against malaria. Despite the small sample size and number of cases in this pilot study, the prognostic associations, particularly with cord blood cytokines, were strong and had C-indices in the range of those observed for models of HIV acquisition [17] and mortality from severe sepsis [18]. Even in the absence of blood-based measurements, a simple model based on factors readily obtainable at the time of delivery (i.e., sex, gravidity, transmission season at delivery, and bed net possession) was able to correctly predict the severe malarial anemia order failure for 63 out of 100 pairs of children – 13 more pairs than chance alone. Further research is needed to externally validate the presented prognostic models of severe malarial anemia risk and to explore the feasibility of incorporating targeted interventions for high-risk individuals into ongoing antimalarial campaigns in developing countries.

The current study had strengths and limitations. In line with the recommendations of the TRIPOD statement [19], candidate predictors were selected to be evaluable both objectively (and thereby testable in validation cohorts) and at the time of delivery (i.e., before children would be subject to interventions that might alter their exposure to *Plasmodium* parasites). Minimizing the potential for detection bias, the outcome of severe malarial anemia was independently diagnosed by clinical teams prior to the selection of potential risk predictors. Another strength of this analysis was that associations between the predictors and outcome were internally validated using a bootstrap resampling approach to investigate and correct for overfitting that could arise from the low event to predictor ratio [13]. Moving forward, it will be important to externally validate the prognostic models for severe malarial anemia in other birth cohorts. In particular, the generalizability of the described prediction models may be limited, and it will be important to validate the models using data from regions with different ecological patterns of malaria transmission (e.g., in regions with low or highly seasonal transmission) where the age of severe malarial anemia onset may be delayed [20]. In addition, future studies should aim to recruit larger sample sizes with greater numbers of severe malaria cases in order to mitigate optimistic model performance estimates. Including more participants will also enable investigations of the potential heterogeneity in the model's discrimination that may exist within clinically relevant sub-groups (e.g., in children exposed to placental malaria in utero).

In regions that are approaching the elimination of malaria, identifying high risk groups based on easily ascertainable and low cost indicators will be particularly relevant, and efforts should be made to enhance the feasibility of malaria screening programs. Specifically, future studies that aim to formally develop prognostic models of life-threatening malaria in childhood should evaluate additional pragmatic predictors, such as parental occupation (e.g., working in the logging industry [21]) and housing conditions (e.g., the presence of window and door screens) [22, 23]. Investigations into the cost-effectiveness of a potential screening program should also consider alternative assays for evaluating children's blood-based biomarkers (e.g., use of dried blood spots [24]) as well as factors that could influence the context-specific protective efficacy of interventional programs (e.g., relative abundance of drug-resistant parasites [25]).

In conclusion, this study provides valuable new evidence that the development of a prognostic model for severe malarial anemia during infancy and early childhood is scientifically plausible. By providing clinicians and families with an objectively estimated probability of a newborn's risk of developing life-threatening complications of infection, personalized medicine approaches have the potential to complement on-going population-wide approaches for malaria prevention.

## Supplementary material

For supplementary material accompanying this paper visit http://dx.doi.org/10.1017/gheg.2017.8. click here to view supplementary material
