# Supplementary material for: Developing a novel risk prediction model for severe malarial anemia
Source: Glob Health Epidemiol Genom. 2017 Sep 11;2:e14. doi: 10.1017/gheg.2017.8 (PMC5732579; doi:10.1017/gheg.2017.8)
Supplement: Supplementary file 1 [file S2054420017000082sup001.docx]

Supplementary Figure S1. Distributions of log_e_-transformed cord blood cytokines and receptors in the MOMS Project (2002 to 2006) birth cohort in Muheza, Tanzania (n=781).

Distributions were estimated at 35 points. Interleukin-4 and interferon-γ distributions were not log_e_-transformed. The spikes at the left side of the distributions arise from the lower detection limit of the assay used. Abbreviations: TNF-RI, tumor necrosis factor-receptor I, TNF-RII, tumor necrosis factor-receptor II.
